# The Effect of Capsaicin-Containing Food on the Swallowing Response

**DOI:** 10.1007/s00455-015-9668-4

**Published:** 2015-11-03

**Authors:** Satoko Shin, Nobumichi Shutoh, Miho Tonai, Naoko Ogata

**Affiliations:** Department of Basic Nursing Sciences, Oita University of Nursing and Health Sciences, 2944-9 Megusuno, Oita, Oita Prefecture 870-1201 Japan; Graduate School of Maritime Sciences, Kobe University, 5-1-1, Fukae-minamimachi, Higashinada-Ku, Kobe, Hyogo Prefecture 658-0022 Japan; Mimihana Clinic, 62 Kuchido, Oita, Oita Prefecture 870-1162 Japan

**Keywords:** Deglutition, Deglutition disorders, Swallowing response, Improvement, Elderly, Food

## Abstract

The purpose of this study was to determine the effect of regular ingestion of capsaicin-containing food that is easily available in everyday life on the latency of the swallowing response (LSR). Pickled Napa cabbage was selected as the food for the present study. One portion (10 g) of pickled Napa cabbage provides 1.5 µg of capsaicin. Participants ingested pickled Napa cabbage (10 g) before every meal for 20 days (days 1–20). LSR was measured pre-intervention (day 0) and post-intervention (day 21). The participants then followed their regular diet, without foods containing red pepper, from day 21 to day 27, and LSR was measured on day 28 (follow-up LSR). Sixteen elderly participants (two male, 14 female; age 81.6 ± 9.39 years) and 10 young participants (all female; age 21.6 ± 0.52 years) participated in the study. The pre-intervention LSR was 2.04 ± 1.60 s in elderly participants and 1.27 ± 0.27 s in young participants. In the elderly group, the post-intervention LSR (day 21) was 1.47 ± 1.05  the follow-up LSR (day 28) was 1.99 ± 1.80 s (*p* = 0.044 and 0.502, respectively, compared to pre-intervention). In the young group, the post-intervention and follow-up LSR values were 1.07 ± 0.20 s and 1.04 ± 0.10 s, respectively (*p* = 0.016 and 0.038, respectively, compared to pre-intervention). Swallowing function was improved by pickled Napa cabbage containing capsaicin, but capsaicin supplementation may need to be maintained to have an ongoing effect.

## Introduction

One of the roles of nurses in an aging society is to extend the healthy life expectancy, i.e., extend the age to which people are healthy, have a purpose in life, and live an independent life. In order for a person to live a self-sustaining life and maintain health, the act of eating is essential, and independent swallowing function is a critical part of this. However, a large proportion of home-dwelling elderly individuals aged ≥65 years experience failure of the pharyngeal phase of swallowing: 12.7 % have reported choking when eating rice, and 17.2 % have reported choking when drinking tea, and the incidence of choking increases with age [1].

The latency of the pharyngeal swallowing response increases with age [2–5], and a pharyngeal delay is a part of normal aging. This increases the risk of aspiration, and approximately 70 % of pneumonia cases in elderly individuals are aspiration pneumonia [6]. More than 96 % of people who died of pneumonia in Japan in 2012 were ≥65 years of age [7]. The age-related decrease in swallowing function is therefore highly likely to endanger life for elderly people, and interventions that prevent or reduce the age-related decrease in swallowing function can extend the healthy life expectancy of elderly people.

One aspect of swallowing function that declines with age is the latency of the swallowing response (LSR) [3, 4, 8]. The LSR is the time from injection to the onset of swallowing. Stimulation of transient receptor potential cation channel (TRPV1), (subfamily V, member 1) increases sensory input to the laryngopharynx. TRPV1 is a nonselective cation channel that is structurally related to members of the TRP family of ion channels, and is expressed in free nerve endings of the superior laryngeal nerve and glossopharyngeal nerve. When stimulation of TRPV1 reaches a certain threshold, and the afferent sensory input to the central pattern generator of the medulla oblongata is activated, motor nerve activity (glossopharyngeal nerve, vagus nerve, hypoglossal nerve) is driven. Input to the central pattern generator that controls swallowing via the corticobulbar projection affects the pharyngeal-stage swallowing response [9]. However, the sensitivities of the laryngopharynx and supraglottic areas are reduced in elderly people [10, 11]. Young adults had a higher number of myelinated fibers in the laryngeal nerves than elderly adults [12]. Therefore, the delay in the swallowing response observed in elderly individuals is thought to be associated with a decrease in sensitivity to stimuli in the laryngopharynx area. Magara et al. showed that the swallowing response time was shortened by electric stimulation of the pharynx for 5 days in healthy adults [13]. We speculate that electrical stimulation of the pharynx increased expression of TRPV1 and increased the afferent sensory input to the central pattern generator for swallowing. Furthermore, we speculate that repetition of this stimulus provoked plasticity of synapses in the cortex medulla.

Capsaicin is a known agonist of TRPV1. Therefore, it may be possible to reduce the LSR by using capsaicin to provide a direct and repeated stimulus to TRPV1. Regular long-term stimulation with capsaicin may also increase the sensory input from the laryngopharynx to the central pattern generator, and thus influence the plasticity of synapses.

Capsaicin contained in red pepper is a pain-producing substance that acts selectively on the primary afferent fibers involved in nociception (predominantly C fibers). Capsaicin caused a dose-related decrease in the contraction time of the tracheal muscle at doses ≥10^−6^ mol l^−1^ [14]. In addition, the latent time of swallowing was longer in elderly individuals with cerebral thrombosis or dementia than in age-matched controls and was reduced by capsaicin (10^−12^–10^−9^ mol ml^−1^) in a dose-dependent manner in patients [15]. These results suggest that capsaicin can reduce the age-related delay in the swallowing response.

Capsaicin is the pungent component of red peppers and can be easily ingested from food. To our knowledge, only three studies have examined the effect of capsaicin ingestion on the swallowing response. Rofes et al. assessed the swallowing response in 33 elderly patients with oropharyngeal dysphagia who ingested nectar boluses containing 150 µM capsaicinoids, and reported that natural capsaicinoids improved the swallowing response of patients with dysphagia, shortening the laryngeal vestibule closure time and enhancing hyoid motion [16]. Goto and colleagues studied 17 healthy male adults and reported that the LSR was shorter 40 min after ingesting capsaicin-containing film (1.5 µg capsaicin per film) than it was before ingesting the film [17]. These two studies indicate that ingestion of capsaicin improves the swallowing response. However, both studies only reported the transient effects of capsaicin on the swallowing response. To influence the age-related decline in the swallowing response, the stimulation would need to be continuous or repeated. Ebihara and colleagues studied the effect of supplementation with capsaicin tablets (1.5 µg per tablet) taken before every meal for 4 weeks on the swallowing response in elderly individuals. The LSR at the end of the 4-week intervention was shorter than that at baseline. This suggests that regular stimulation by capsaicin may be effective at reducing the LSR in older adults [18]. Taken together, these studies suggest that capsaicin ingestion can effectively improve swallowing response function.

However, among those studies, only the research of Rofes et al. used capsaicin in the form of food, and the other two studies used capsaicin tablets or films, which were not in the form of food. Regular ingestion of these forms of capsaicin might be hard to adhere to due to high cost or poor availability. To encourage continued and repeated stimulation, the method of ingesting capsaicin must be easy for elderly individuals to incorporate into everyday life. The purpose of this study was to determine the effect of regular ingestion of capsaicin-containing food that is easily available in everyday life on the LSR. The results may inform interventions or guidelines designed to prevent the age-related decrease in swallowing function.

## Methods

This study was performed in two steps. In step one, the capsaicin-containing food for ingestion was selected. In step two, the effect of regular ingestion of this capsaicin-containing food on swallowing function was assessed.

### Selection of the Capsaicin-Containing Food for Ingestion

The results of previous studies indicate that ingestion of 1.5 µg of capsaicin in the form of tablets or film sheets can promote the swallowing response [17, 18]. Based on these results, the intake amount of capsaicin in one meal was set at 1.5 µg in the present study. Three capsaicin-containing foods prepared using different cooking methods were considered: kimchi, pickled Napa cabbage, and fried burdock root. The capsaicin concentration of each food was measured by high-performance chromatography and is shown in Table 1. The capsaicin concentration of the pickled Napa cabbage was closest to the capsaicin concentration used in previous studies, and hence, pickled Napa cabbage was selected as the food for the present study.

**Table 1:** Capsaicin concentration

Food	Capsaicin content (mg per 100g)
Kimichi	0.6
Fried budock root	0.1
Japanese pickled Napa cabbage manufacturer A	0.01
Japanese pickled Napa cabbage manufacturer B	0.009
Japanese pickled Napa cabbage manufacturer C, sample 1	0.011
Japanese pickled Napa cabbage manufacturer C, sample 2	0.025
Japanese pickled Napa cabbage manufacturer C, sample 3	0.012
Japanese pickled Napa cabbage manufacturer C, sample 4	0.015
Japanese pickled Napa cabbage manufacturer C, sample 5	0.012

Pickled Napa cabbages from three different manufacturers were considered (manufacturer A, B, and C). The capsaicin concentration of pickled Napa cabbages from each of the manufacturers was measured by high-performance chromatography. All three had similar capsaicin concentration (Table 1). Manufacturer C was selected because it had a clear production method and was able to supply large quantities of the product at a time. Five samples were selected at random from the pickled Napa cabbages contained in a single vessel at manufacturer C, and the capsaicin concentration of each sample was measured by high-performance chromatography. The capsaicin concentration of the five samples is shown in Table 1. The average (mean ± SD) capsaicin concentration of the five pickled Napa cabbages was 0.015 ± 0.006 mg per 100 g. Therefore, 10 g of pickled Napa cabbage provides 1.5 µg of capsaicin, which is in accordance with the dose used in previous studies.

The Napa cabbage used in this study was pickled in the usual way by the manufacturer (Beppu Tsukemono Co. Ltd.). In brief, the cabbage was salted for 12 days then mixed with two or three slices of red pepper in a soup for seasoning, and left to sit for 3 days. The pickled cabbage was semi-vacuum-sealed in vinyl bags (200 g cabbage per bag) and refrigerated.

### Participants

Young adults were recruited through a snowball sampling research method. All young adult participants attended the undergraduate university at which the author is affiliated. All young adult participants were healthy adults aged ≥20 years old, and no participant had a history of dysphagia. Older adults were recruited through two long-term health care facilities. No participants had any history of dysphagia or aspiration pneumonia, and all could independently prepare and eat a meal. The ethics and safety committee of the author’s institution approved this study (approval number 477). Written informed consent was obtained from all participants or their families before enrollment in the study. In each case, staff at the health care facility determined if the participant was capable of providing informed consent, or if the informed consent should be obtained from the participant’s family.

### Outcome Measure

The LSR was assessed using simple swallowing provocation time (S-SPT) [19] and was defined as the time from stimulation of the pharyngeal region to the onset of the swallowing response. The onset of the swallowing response was identified by visual observation of the characteristic laryngeal movement. A syringe containing glucose solution (0.4 ml) was connected to the inlet of a nasoesophageal feeding tube [5 Fr (1.7 mm) outer diameter, atom medical] filled with 5 % glucose, then the tube was inserted 13–14 cm from the nasal cavity to the oropharynx while the participant was in a supine position. The catheter position was confirmed macroscopically using light from the oral cavity. Then, the glucose solution (0.4 ml) was injected at the end of expiration. The syringe was kept out of sight of the subject. At the same time, the larynx was recorded at 30 frames per second using a digital high-definition video camera (HC-V300 M, Panasonic) to allow the onset of the swallowing response to be identified. Only the neck was recorded. The face was not recorded, meaning that the identity of the subject could not be determined from the video recording. The person performing the data analysis was blinded as to the subject group (young, elderly) and time point of the recording. The procedure was carried out three times with break of 1 min between repetitions. The time from glucose infusion to laryngeal movement was measured offline using Windows Live Movie Maker (Microsoft Corporation) with a frame step of 0.01 s. The reported LSR is the average of the two shortest times. All S-SPT measurements were performed by one experimenter to reduce variability.

### Experimental Protocol

The first S-SPT was measured prior to the intervention (pre-intervention; day 0). The supply of pickled Napa cabbage was then given to the person who prepared the meals at the health care facility or the participant’s home. They were instructed to discard the red peppers and the soup and then lightly squeeze the pickled cabbages to remove the juice. Participants were instructed to ingest about 10 g of the pickled cabbage (approximately one tablespoon) just before the start of each meal. These instructions were provided orally and in writing. Participants began the intervention the morning after the pre-intervention S-SPT and continued for 20 days (days 1–20). The post-intervention S-SPT was performed on day 21. The participant then followed their regular diet, without foods containing red pepper, from day 21 to day 27 and a follow-up S-SPT was performed on day 28. All S-SPT measurements were performed at least 1 h after the previous meal.

### Data Analysis

**Fig. 1:**
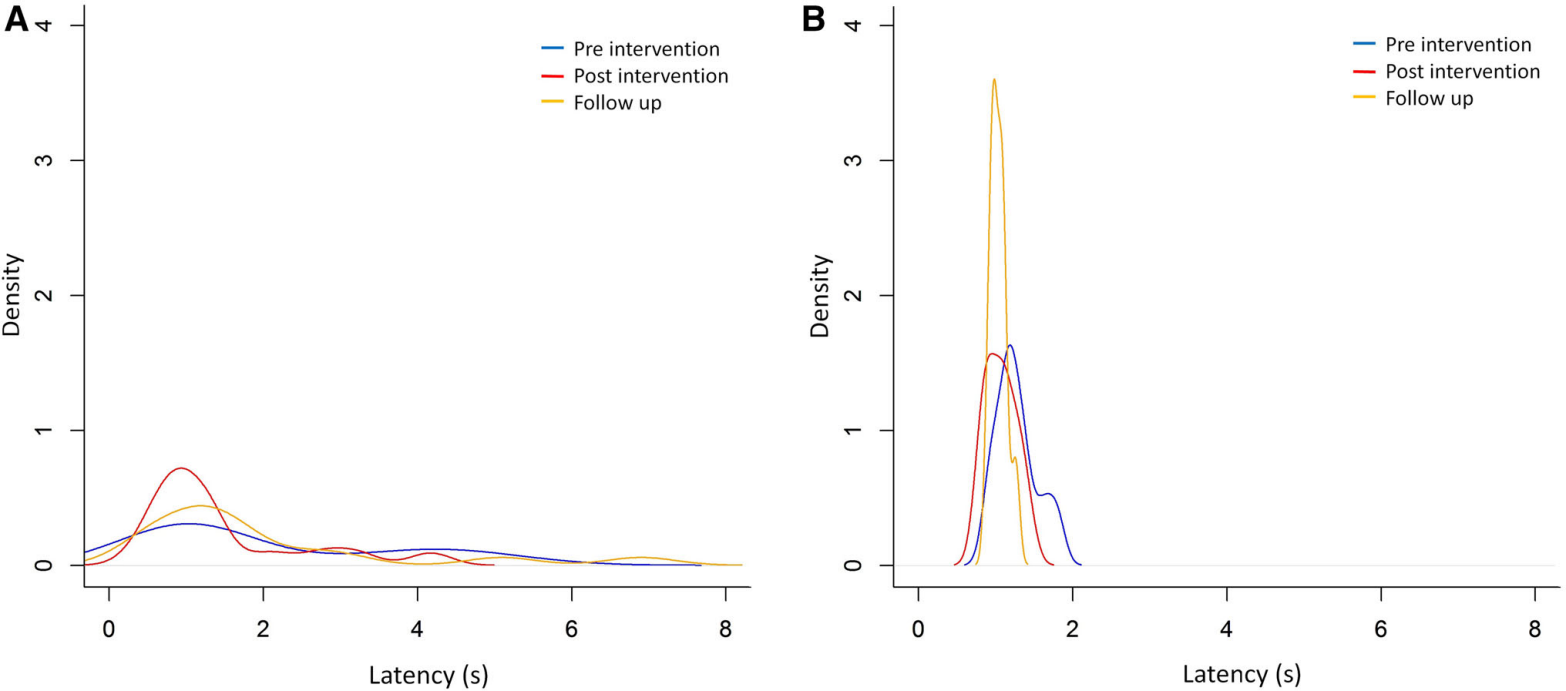
Probability density estimation of the latency of the swallowing response in elderly (**a)** and young (**b)** participants at day 0 (pre intervention; *blue* *line*), day 21 (post intervention; *red* *line*), and day 28 (follow up; *orange line*)

Data were analyzed using R3.0.3 (GNU R, R Development Core Team), Excel 2010 (Microsoft Excel 2010, Microsoft Corporation), and SPSS version 21 (IBM SPSS Statistics). Density estimation (implemented in R 3.0.3) was used to evaluate the distribution of the LSR in each group at each time point (pre-intervention, post-intervention, follow-up; Fig. 1). The null hypothesis for multivariate normality was rejected for both groups (*p* < 0.001 for the elderly group, and *p* = 0.001 for the young group). Therefore, statistical analysis was performed using the bootstrap method, which does not require assumption of normality. Multiple comparisons of means with a control were performed, and *p* values were adjusted using the Bonferroni method. All data are presented as mean ± SD.

## Results

Sixteen elderly participants (two male, 14 female; age 81.6 ± 9.39 years) and 10 young participants (all female; age 21.6 ± 0.52 years) participated in the study. The elderly participants had various medical conditions, including hypertension (*n* = 11), respiratory disease (*n* = 4), stroke (*n* = 4), gastrointestinal disease (*n* = 2), diabetes (*n* = 3), and other conditions (*n* = 8). The young participants had no medical conditions. The follow-up LSR measurement was not performed for one participant in the elderly group. Therefore, comparisons that involve the follow-up time point in the elderly group include only 15 participants.

**Fig. 2:**
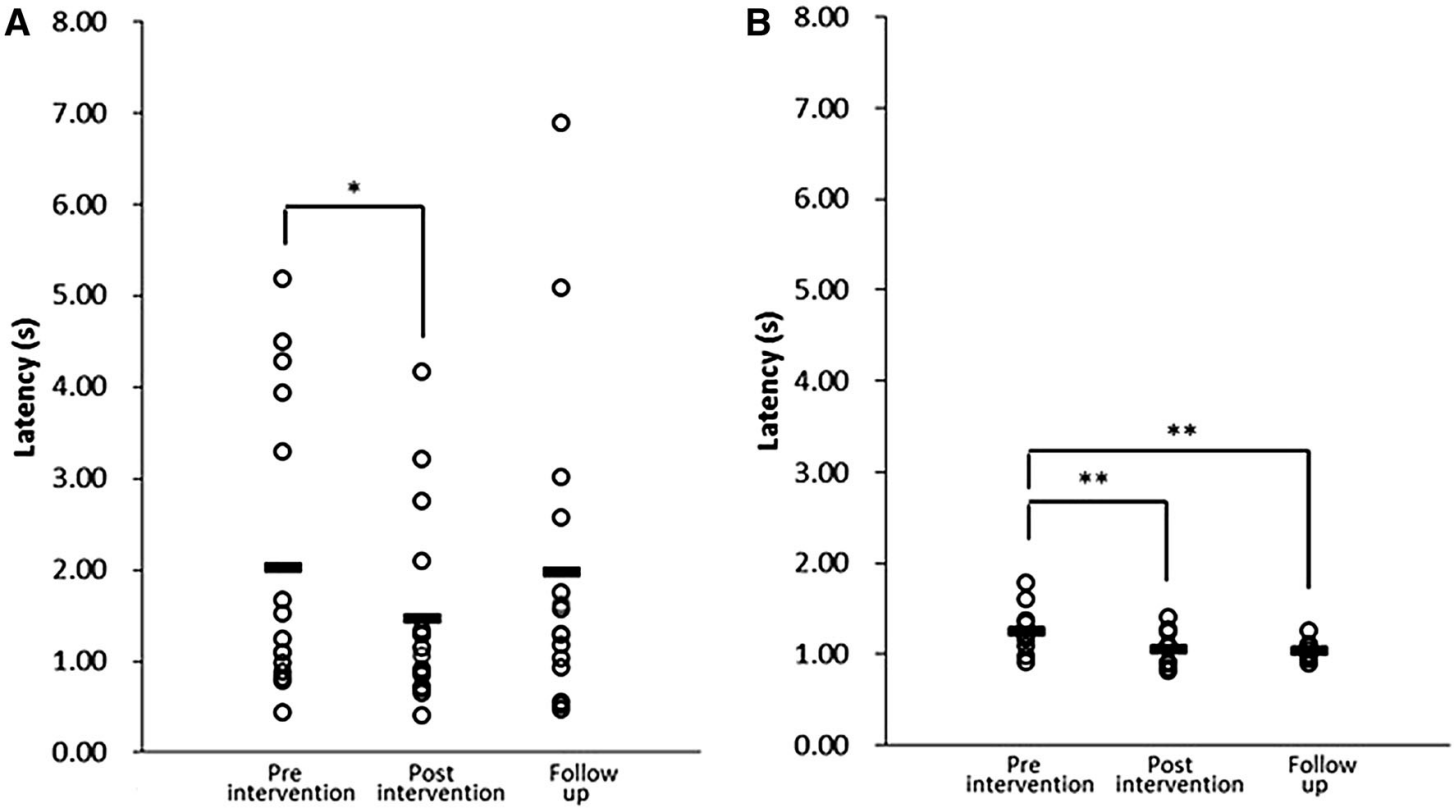
The latency of the swallowing response in elderly (**a)** and young (**b)** participants. *Horizontal* *bars* represent the mean value (*n* = 16 at pre- and post-intervention time points in the elderly group, *n* = 15 at the follow-up time point in the elderly group, and *n* = 10 at each time point in the young group). *Asterisks* indicate a significant difference between the time points (*p < 0.10, **p < 0.05). Statistical analyses were performed using a bootstrap test

The pre-intervention LSR was 2.04 ± 1.60 s in the elderly group and 1.27 ± 0.27 s in the young group (Table 2; *p* = 0.044, two-sample bootstrap test). In the elderly group, the post-intervention LSR was 1.47 ± 1.05 s, and the follow-up LSR was 1.99 ± 1.80 s. The post-intervention LSR was significantly lower than the pre-intervention LSR (*p* = 0.044, bootstrap test), but the follow-up LSR was not significantly different from the pre-intervention LSR (*p* = 0.502, Table 2; Fig. 2). In the young group, the post-intervention LSR was 1.07 ± 0.20 s, and the follow-up LSR was 1.04 ± 0.10 s. The LSR values at both of these time points were significantly lower than the pre-intervention LSR (*p* = 0.016 for post-intervention LSR and *p* = 0.038 for follow-up LSR, bootstrap test; Table 2; Fig. 2).

**Table 2:** Latency of the swallowing response in elderly and young groups

	Elderly group (*n* = 16; age 81.6 ± 9.39 years)	Young group (*n* = 10; age 21.6 ± 0.52 years)
Pre-intervention LSR (s)	2.04 ± 1.60	1.27 ± 0.27
Post-intervention LSR (s)	1.47 ± 1.05*	1.07 ± 0.20**
Follow-up LSR (s)	1.99 ± 1.80^1^	1.04 ± 0.10**

*LSR* the latency of the swallowing response

Bootstrap test (vs pre-intervention LSR for each group): * p < 0.10, ** p < 0.05

^1^n=15

Before the intervention there was large inter-individual variation in the LSR of elderly individuals. However, the LSR measured immediately after the 20-day intervention had less inter-individual variation, and was concentrated around 1 s (Fig. 2). In the young participants, the inter-individual variation in the LSR before the invention was small. However, the inter-individual variation was still reduced slightly from the pre- to the post-intervention time points (Fig. 2).

## Discussion

The purpose of this study was to determine the effect of regular ingestion of capsaicin-containing food that is easily available in everyday life on the LSR. Our findings indicate that regular ingestion of a capsaicin-containing food improved the swallowing response in elderly and young people.

### Training Effect on S-SPT

In this study, S-SPT was used to evaluate swallowing response and was performed on three occasions in each participant (pre-intervention, post-intervention, and follow-up). The interval between measurements was long, either 7 or 20 days. On each occasion, the S-SPT was measured three times, with an interval of about 1 min between consecutive water injections. Each injection of water (i.e., each stimulation) lasted approximately 1 s. The syringe was kept out of sight of the subject. Together, this indicates that it is unlikely that the repeated measurements affected the S-SPT via a training effect. However, there is a need to consider this possibility in the future.

### Baseline Swallowing Response in Elderly Participants

In this study, the LSR of elderly participants before the intervention was significantly higher than that of young participants. This decrease in swallowing function is likely related to aging. In a large study of 220 subjects aged from 20 to 80 years, Teramoto [3] showed that swallowing-induced threshold volume and LSR increased with age. Similarly, Ameya et al. [8] showed significant prolongation of the pharyngeal transition time and laryngeal elevation delay time with aging. The LSR of the elderly group in this study was 2.04 ± 1.60 s. Responses to the S-SPT are classified as abnormal if they occur after 3 s after bolus injection [20]. Therefore, the LSR of the healthy elderly participants in this study was within the range expected for normal swallowing function. However, the significant difference between the elderly and young groups suggests that the elderly participants had a lower residual capacity than the young participants.

### The Effect of Pickled Cabbage Ingestion on the LSR

After 20 days of taking pickled Napa cabbage before each meal, the LSR of the elderly participants was significantly shorter than it was before the intervention. The LSR of the young participants also shortened significantly after the intervention. This indicates that the ingestion of pickled Napa cabbage before each meal for 20 days improved the swallowing response regardless of the age of the participant.

In addition, after 20 days of taking pickled Napa cabbage before each meal, inter-individual variation in the LSR of the elderly participants was reduced, and the LSR was concentrated around 1 s. In the same way, the inter-individual variation of the young was still reduced slightly. This indicates that the ingestion of pickled Napa cabbage before each meal for 20 days improved the swallowing response regardless of the baseline swallowing function.

In this study, we measured the post-intervention LSR on day 21, i.e., the day after the last day of the intervention, rather than immediately after ingestion of the final meal with pickled Napa cabbage, and evaluated the change in swallowing response function by comparing this LSR to that measured before the intervention. Taking the measurement on day 21 meant that at least 15 h had passed since the last ingestion of the pickled Napa cabbage. Goto et al. [17] reported that the LSR measured 40 min after the ingestion of capsaicin-containing films was larger than that measured before the ingestion of capsaicin-containing films. The LSR measured ≥15 h after the last ingestion of capsaicin would reflect the swallowing response function without the influence of any transient effects of capsaicin ingestion.

Rofes et al. [16] reported that capsaicinoids exerted a strong therapeutic effect by enhancing afferent sensory input through stimulation of oropharyngeal TRPV1 channels, shortening laryngeal vestibule closure time, upper esophageal sphincter opening time, and the time to maximal vertical extension of hyoid and larynx. This is consistent with the shortening of the LSR observed in this study. A further feature of the present study is that it involved repeated delivery of capsaicin. In other words, repeated stimulation over the 20 days is considered to have had an influence on the plasticity of synapses.

Our results suggest that, even in elderly individuals with swallowing function within the normal range, there is a possibility that swallowing function can be improved by regularly ingesting capsaicin-containing foods. These results support those of a previous study by Ebihara et al. [18] in which swallowing function improved after elderly individuals took capsaicin trochsci supplements for 4 weeks.

The improvement in swallowing function from pre- to post intervention occurred in young as well as in elderly participants (Table 2; Fig. 2). This suggests that there is room for improvement in swallowing function even in young individuals, and it may be possible for early interventions to maintain a high level of swallowing function in addition to preventing age-related declines in function.

### The Duration of the Intervention

The duration of the intervention was less than that in the study of Ebihara et al. [18], and our results show that regular capsaicin ingestion influenced swallowing function even over a period as short as 20 days. In the elderly participants, the inter-individual variation in LSR reduced from pre- to post intervention, and LSR clustered around 1 s at the post-intervention time point (see Fig. 2). In the young participants, LSR also clustered around 1 s. It might be possible that ingestion of capsaicin-containing food can improve the LSR to around 1 s. However, it is necessary to consider the cause of the inter-individual variation in the elderly participants, and whether this can be improved by capsaicin supplementation alone, independently of supplementation with vegetables such as Napa cabbage or other forms of seasoning.

### Maintenance of Capsaicin-Induced Effects

Seven days after the end of the intervention, the LSR of the elderly participants had returned to the pre-intervention baseline. This suggests that capsaicin must be taken continuously in order to prevent deterioration in function. By contrast, at 7 days after the intervention, the LSR of young participants was similar to that measured immediately after the intervention. This indicates that the capsaicin-induced functional improvement was maintained for at least a week in the young participants. If regular capsaicin supplementation is introduced as a preventive action from a young age, the high swallowing capacity might be preserved, and it may be possible to take breaks of about 1 week in the supplementation rather than take continuous supplementation.

### Advantages of Capsaicin Ingestion via Food Products

There has been only one previous study of the effect of medium-term capsaicin supplementation on swallowing function [18]. In the present study, capsaicin was provided in the form of a food that is regularly eaten as part of the typical Japanese diet, and therefore has the advantage of being accessible and possible to enjoy with a meal. This may make it easier to adhere to continuous capsaicin intake. In addition, the required intake is only about one tablespoon per meal.

### Study Limitations

We have attributed the effects of the intervention in this study to capsaicin. However, as the participants ingested pickled Napa cabbage, there is a need for further study to clarify whether the observed effects were due to capsaicin, or to components in the food other than capsaicin. Future studies should also examine methods of ingestion that are easy to introduce into everyday life, and determine the optimal duration of intervention and number of times of day for supplement intake. This study did not include a control group. Therefore, it is not possible to compare swallowing function after 20 days between participants who ingested a capsaicin-containing food and participants who did not. This study involved repeated measurements. Using videofluoroscopic examination of swallowing (VFS), it is possible to directly observe the movements of the pharyngeal muscles. However, VFS involves risk of radiation exposure, and measurements take a long time. Therefore, it was not possible to use VFS for evaluation in this study. In future studies that aim to evaluate the involuntary swallowing reaction in detail, it will be necessary to use VFS.

## Conclusion

The aim of this study was to determine the effect of pickled Napa cabbage supplementation on swallowing function in elderly individuals. Participants ingested pickled Napa cabbage before each meal over a 20-day period. We found that the swallowing function (LSR) of healthy elderly participants was within the normal range but was lower than that of young participants, suggesting lower residual capacity. Ingestion of pickled Napa cabbage before each meal for 20 days improved the LSR in both groups, suggesting that swallowing function might be improved by capsaicin supplementation regardless of the baseline state and the age of the participant. The improvement in LSR was not maintained at 1-week post intervention, suggesting that capsaicin supplementation needs to be maintained to have an ongoing effect on swallowing function.

## References

[1] Kamakura Y, Okamoto K, Sugimoto S (1998). Swallowing and its correlates in the aged living at home. Sogo Rehabil.

[2] Ohmae Y, Sugiura M, Mogitate M (2003). Swallowing function in the very old (85 + years): changes in swallowing physiology with normal aging. J Jpn Bronchoesophagol Soc.

[3] Teramoto S (2002). Age-related changes of swallowing reflex in human. Jpn J Chest Dis.

[4] Shaker R, Ren J, Zamir Z, Sarna A, Liu J, Sui Z (1994). Effect of aging, position, and temperature on the threshold volume triggering pharyngeal swallows. Gastroenterology.

[5] Okamura H, Inaki S, Mori T, Fukui K, Aibara R (1991). Pharyngeal swallowing function in the elderly-videofluoroscopic observations. J Jpn Bronchoesophagol Soc.

[6] Teramoto S, Fukuchi Y, Sasaki H, Sato K, Sekizawa K, Matsuse T (2008). High incidence of aspiration pneumonia in community- and hospital-acquired pneumonia in hospitalized patients: a multicenter, prospective study in Japan. J Am Geriatr Soc.

[7] Portal site of official statistics of Japan. Leading causes of death by sex and age, Japan, vol. 1: 5–17, http://www.e-stat.go.jp/SG1/estat/ListE.do?lid=000001108739 (2014). Accessed 18 Nov 2014.

[8] Ameya M, Nishikubo K, Mise K, Motoyoshi K, Hyodo M (2006). Dysphagia associated with advancing age. Jibi to Rinsho.

[9] Umezaki T (2007). Neural organization of swallowing. High Brain funct Res.

[10] Aviv JE, Martin JH, Jones ME, Wee TA, Diamond B, Keen MS, Blitzer A (1994). Age-related changes in pharyngeal and supraglottic sensation. Ann Otol Rhinol Laryngol.

[11] Aviv JE (1997). Effects of aging on sensitivity of the pharyngeal and supraglottic areas. Am J Med.

[12] Tiago R, Pontes P, Brasil OC (2007). Age-related changes in human laryngeal nerves. Otolaryngol Head Neck Surg.

[13] Magara J, Taniguchi H, Hayashi H, Takeishi R, Tsujimura T, Hori K, Inoue M (2014). Possible neuroplasticity of swallow related function by pharyngeal electrical stimulation. J Jpn Soc Stomatognathic Funct.

[14] Aizawa H, Miyazaki N, Tomooka M, Sigematsu N, Ejima T (1988). Substance P increases acetylcholine release from vagal nerve terminals. Jpn Soc Allergol.

[15] Ebihara T, Sekizawa K, Nakazawa H, Sasaki H (1993). Capsaicin and swallowing reflex. Lancet.

[16] Rofes L, Arreola V, Martin A, Clave P (2013). Natural capsaicinoids improve swallow response in older patients with oropharyngeal dysphagia. Gut.

[17] Goto T, Murata N, Maekawa K, Kanda Y, Kobayashi Y, Mori T, Miyawaki T, Egusa M (2013). Facilitatory effect of an applying capsaicin-containing film on initiation of swallowing reflex. Jpn J Dysphagia Rehabil.

[18] Ebihara T, Takahashi H, Ebihara S, Okazaki T, Sasaki T, Watando A, Nemoto M, Sasaki H (2005). Capsaicin troche for swallowing dysfunction in older people. J Am Geriatr Soc.

[19] Teramoto S, Fukuchi Y (2000). Detection of aspiration and swallowing disorder in older stroke patients: simple swallowing provocation test versus water swallowing test. Arch Phys Med Rehabil.

[20] Teramoto S, Matsuse T, Matsui H, Ohga E, Saitoh E, Ishii T, Tomita T, Nagase T, Fukuchi Y, Ouchi Y (1999). The simple swallowing provocation test as a means of screening for swallowing disorders: a comparison with the water swallowing test. J Jpn Respir Soc.

